# Monitoring and modelling marine zooplankton in a changing climate

**DOI:** 10.1038/s41467-023-36241-5

**Published:** 2023-02-02

**Authors:** Lavenia Ratnarajah, Rana Abu-Alhaija, Angus Atkinson, Sonia Batten, Nicholas J. Bax, Kim S. Bernard, Gabrielle Canonico, Astrid Cornils, Jason D. Everett, Maria Grigoratou, Nurul Huda Ahmad Ishak, David Johns, Fabien Lombard, Erik Muxagata, Clare Ostle, Sophie Pitois, Anthony J. Richardson, Katrin Schmidt, Lars Stemmann, Kerrie M. Swadling, Guang Yang, Lidia Yebra

**Affiliations:** 1 Integrated Marine Observing System, Hobart, Tasmania Australia; 2 Global Ocean Observing System, International Oceanographic Commission, UNESCO, Paris, France; 3 Cyprus Subsea Consulting and Services C.S.C.S. ltd, Lefkosia, Cyprus; 4 Plymouth Marine Laboratory, Prospect Place, The Hoe, PL1 3DH Plymouth, UK; 5 North Pacific Marine Science Organization (PICES), 9860 West Saanich Road, V8L 4B2 Sidney, BC Canada; 6 CSIRO Oceans & Atmosphere, Hobart, Tasmania Australia; 7 College of Earth, Ocean, and Atmospheric Sciences, Oregon State University, 104 CEOAS Admin Bldg., Corvallis, OR 97330 USA; 8 US Integrated Ocean Observing System (US IOOS), NOAA, Silver Spring, MD USA; 9 Alfred Wegener Institute Helmholtz Centre for Polar and Marine Research, Section Polar Biological Oceanography, Am Handelshafen 12, Bremerhaven, Germany; 10 School of Mathematics and Physics, University of Queensland, St. Lucia, QLD Australia; 11 CSIRO Oceans and Atmosphere, Queensland Biosciences Precinct, St Lucia, 4067 Australia; 12 Evolution and Ecology Research Centre, University of New South Wales, Sydney, NSW Australia; 13 Gulf of Maine Research Institute, 350 Commercial St, Portland, ME 04101 USA; 14 Mercator Ocean International, 2 Av. de l’Aérodrome de Montaudran, 31400 Toulouse, France; 15 Faculty of Science and Marine Environment, Universiti Malaysia Terengganu, 21030 Kuala Nerus, Terengganu Malaysia; 16 Institute of Oceanography and Environment, Universiti Malaysia Terengganu, 21030 Kuala Nerus, Terengganu Malaysia; 17 The Marine Biological Association (MBA), The Laboratory, Citadel Hill, Plymouth PL1 2PB UK; 18 Sorbonne Université, Centre National de la Recherche Scientifique, Laboratoire d’Océanographie de Villefranche (LOV), Villefranche-sur-Mer, France; 19 Research Federation for the Study of Global Ocean Systems Ecology and Evolution, FR2022/Tara Oceans GOSEE, 75016 Paris, France; 20 Institut Universitaire de France, 75231 Paris, France; 21 Universidade Federal de Rio Grande - FURG - Laboratório de Zooplâncton - Instituto de Oceanografia, Av. Itália, Km 8 - Campus Carreiros, 96203-900 Rio Grande, RS Brazil; 22 Centre for Environment, Fisheries and Aquaculture Centre (Cefas), Pakefield Road, Lowestoft, NR330HT UK; 23 School of Geography, Earth and Environmental Sciences, University of Plymouth, Plymouth, PL4 8AA UK; 24 Institute for Marine and Antarctic Studies & Australian Antarctic Program Partnership, University of Tasmania, Hobart, Tasmania Australia; 25 Key Laboratory of Marine Ecology and Environmental Sciences, Institute of Oceanology, Chinese Academy of Sciences, 7 Nanhai Road, Qingdao, 266071 PR China; 26 Centro Oceanográfico de Málaga (IEO, CSIC), Puerto Pesquero s/n, 29640 Fuengirola, Spain

**Keywords:** Ecosystem ecology, Marine biology, Carbon cycle, Marine chemistry, Environmental health

## Abstract

Zooplankton are major consumers of phytoplankton primary production in marine ecosystems. As such, they represent a critical link for energy and matter transfer between phytoplankton and bacterioplankton to higher trophic levels and play an important role in global biogeochemical cycles. In this Review, we discuss key responses of zooplankton to ocean warming, including shifts in phenology, range, and body size, and assess the implications to the biological carbon pump and interactions with higher trophic levels. Our synthesis highlights key knowledge gaps and geographic gaps in monitoring coverage that need to be urgently addressed. We also discuss an integrated sampling approach that combines traditional and novel techniques to improve zooplankton observation for the benefit of monitoring zooplankton populations and modelling future scenarios under global changes.

## Abstract (web summary)

Zooplankton are a critical link to higher trophic levels and play an important role in global biogeochemical cycles. This Review examines key responses of zooplankton to ocean warming, highlights key knowledge and geographic gaps that need to be addressed, and discusses how better use of observations and long-term zooplankton monitoring programmes can help fill these gaps.

## Introduction

Zooplankton are a critical component of marine ecosystems. They are an important pathway for energy transfer between primary producers and higher trophic levels such as fish, seabirds and marine mammals^1–4^, and they influence oceanic biogeochemical cycles through direct and indirect feedback loops^5–12^. Decades of laboratory and field investigations into zooplankton physiology, community composition, and distribution, have shown the sensitivity of zooplankton to the changing ocean – climate-induced poleward shifts in the distribution of some zooplankton had already been observed by the 1960s^13–15^. However, advances in observational data coverage have largely been focussed on the Northern Hemisphere with inconsistent patterns. Changes in zooplankton are altering biogeochemical cycling, energy transfer pathways and the ecosystem services humankind receives from the ocean, but how and where zooplankton will moderate or amplify these processes, particularly under future ocean conditions, has received little attention.

In this Review, we (1) outline the recent and projected changes in global and regional climatic conditions that can impact zooplankton, (2) review climate-driven changes in zooplankton ecological dynamics and their role in ecosystem functioning at local and regional scales, highlighting similarities and discrepancies in trends, (3) highlight existing limitations of zooplankton modelling and discuss how better use of observations can fill some of these gaps, (4) identify current long-term zooplankton monitoring programmes globally, highlighting data availability and gaps in coverage, and lastly (5) look towards the future of global zooplankton research, where we draw on our existing knowledge to advocate for an integrated approach to zooplankton research and monitoring that will link to global needs.

Here, we consider all zooplankton groups, but we focus on net-caught zooplankton, which include copepods, ichthyoplankton (fish eggs and larvae), euphausiids and salps. These groups were selected because they represent the dominant metazoan groups with key ecological and biogeochemical significance and provide ecosystem services such as nutrient recycling and carbon sequestration, as well as supporting fish stocks and fisheries. Additionally, these groups are the most broadly studied geographically, providing the opportunity for a comprehensive global review. Although not the focus, we acknowledge the importance of other zooplankton groups, especially the understudied protists, amphipods, chaetognaths, pteropods and other gelatinous groups (for example, jellyfish, ctenophores and siphonophores), and when possible, we include them in the text.

## Recent and projected climate-driven environmental changes

**Fig. 1:**
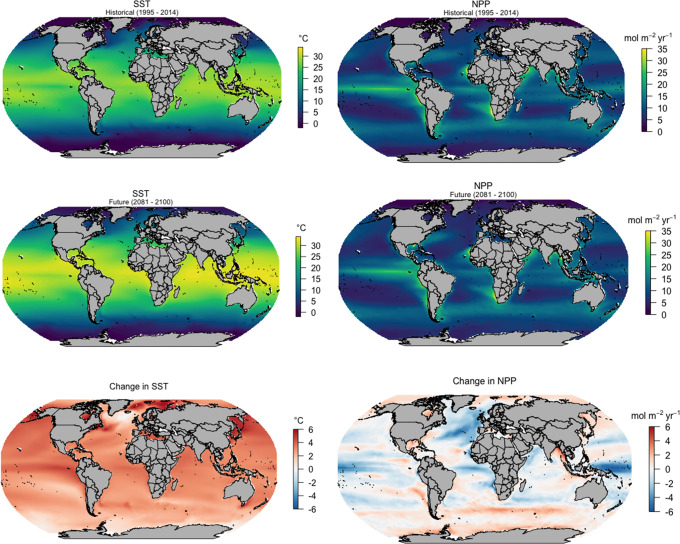
Predicted changes in sea surface temperature (SST) and net primary productivity (NPP) for the global ocean. Multi-model mean projections for SST and NPP from 10 CMIP6 Earth system models for the historical period (1995–2014), future (2081–2100) and the change in SST and NPP by 2081–2100 relative to 1995–2014 based on SSP5–8.5. Publicly available datasets were analysed in this review. The 10 CMIP6 Earth system models used were ACCESS-ESM1.5, CESM2, CESM2-WACCM, CNRM-ESM2-1, GFDL-ESM4, IPSL-CM6A-LR, MIROS-ES2L, MPI-ESM1.2HR, NorESM2-LM and UKESM1-0-LL. This data can be found at: https://esgf.llnl.gov/.

Climate varies on local and regional scales, and patterns are not necessarily equal across all oceanic basins. For example, sea surface temperature (SST) is increasing across all basins, but its rate of increase varies regionally, with projections showing the North Atlantic warming at a much faster rate than the Southern Ocean (Fig. 1). Net primary production (NPP) on the other hand is projected to generally increase towards the poles and decrease toward the equator, but with considerable regional variation (Fig. 1). Future warming leads to enhanced ocean stratification, and this process impacts regional phytoplankton productivity^16,17^. Uncertainties in projected NPP have increased in the latest Coupled Model Intercomparison Project (CMIP6) compared with those of earlier models from CMIP5 (and despite better agreement with the historical records), as more realism has been included in the models, which is likely to increase in the future^17^. Whilst changes in SST and NPP, as well as interannual fluctuations in large-scale climate oscillations (see Box 1) are occurring simultaneously, and influencing zooplankton ecology and ecosystem function, we focus our Review on the impacts of ocean warming and not the regionally variable impacts of changing phytoplankton quality and quantity. Below we untangle some of these climate-driven environmental impacts on zooplankton at local or regional scales.

Box 1 Definition of climate events

**Table:** 

Marine heatwave	Persistent anomalously warm waters for at least 5 consecutive days.
El Niño Southern Oscillation	**ENSO** is the strongest year-to-year climate variability on the planet, originating in the equatorial Pacific Ocean through coupled ocean-atmosphere interactions. ENSO manifests itself in anomalous surface warming or cooling that tends to peak in boreal winter.
El Niño	**El Niño** is the warm phase of ENSO, characterised by anomalous surface warming and weaker trade winds in the equatorial Pacific Ocean.
North Atlantic Oscillation (NAO)	**NAO** is the primary model of internal atmospheric variability in the North Atlantic characterised by a north-south dipole of alternating sea level pressure anomalies between the subtropics and high latitudes.
Southern Annular Mode (SAM)	**SAM** is the leading mode of large-scale atmospheric variability in the Southern Hemisphere, characterised by an anomalous pressure centre over Antarctica and zonally symmetric pressure anomaly of opposite sign at midlatitudes. The positive and negative phases of the SAM are respectively associated with a poleward and equatorward displacement of the midlatitude westerly winds.

Sources for definition: Marine heatwave^51^, ENSO^181^, El Niño^181^, NAO^181^, SAM^181^

## Untangling climate effects on zooplankton ecology and ecosystem function

Multiple climate-induced stressors such as ocean warming and ocean acidification favour some taxa over others and thereby modulate zooplankton community structure. Long-term time series have proved invaluable for elucidating these patterns, particularly in highlighting three ‘universal’ responses to warming despite multiple interacting stressors. These include shifts in phenological timing, typically towards earlier seasonal occurrence of spring or summer species and later occurrence of autumn species^18,19^, poleward shifts in geographical range^20^, and shifts towards smaller size in warmer conditions^21–23^. These changes can have cascading impacts on the efficiency of the biological carbon pump (the biologically driven sequestration of atmospheric carbon dioxide into the ocean’s interior) and transfer efficiency throughout food webs, including desynchronising ecological interactions (for example, between predator and prey), ultimately threatening ecosystem function and services, including fisheries^24^. In the following subsections we discuss similarities and discrepancies in observed patterns and trends by oceanic regions, explore the connectivity between zooplankton ecology and ecosystem function, and highlight key knowledge gaps that drive these uncertainties.

### Zooplankton phenology

Phenology is highly responsive to a species’ temperature sensitivity^18,25,26^, thermal optima^27^ and adaptation rates^28^. In the Intergovernmental Panel on Climate Change (IPCC) Sixth Assessment Report, a systematic review of marine phenology studies suggested that zooplankton timing is moving earlier and responding faster compared to that observed for other marine animals^29^. For example, in the Subarctic Pacific Ocean, ocean warming has caused the biomass of the dominant copepod, *Neocalanus plumchrus*, to peak 14 days earlier per decade or 73 days earlier per increase in °C^30^. In the Central North Sea region of the Atlantic Ocean, the timing of peak biomass for copepods, decapods, echinoderm larvae and other meroplankton and holozooplankton all occurred earlier, ranging from 2.2 to 10 days earlier per decade, or 11.1 to 52 days earlier per increase in °C^18,31,32^. In Narragansett Bay, an inlet of the North Atlantic Ocean, the biomass of the comb jelly, *Mnemiopsis leidyi*, peaked 11.1 days earlier per decade or 49.2 days earlier per increase in °C, although there was no statistically significant change in the phenology of one of their dominant copepod prey, *Acartia tonsa*, over the same time period^33^. In the Gironde estuary in southwest France, zooplankton phenology moved earlier over the past three decades, and this was also apparent in the arrival of fish species into the estuary^34^. Most of the time-series studies on zooplankton phenology have occurred in the Northern Hemisphere, and less is known of the tropics and the Southern Hemisphere^32,35^, highlighting a critical knowledge gap that needs urgent address. However, long-term monitoring of the pteropod *Limacina helicina antarctica* at the Palmer Station Antarctica Long Term Ecological Research (LTER) project shows that the phenology of this pteropod along the Western Antarctic Peninsula has remained relatively stable despite considerable environmental variability^36^.

### Zooplankton range shifts

Under a warming environment, species have generally shifted polewards and/or to deeper layers to maintain their core within their optimum water temperature ranges^32,37,38^. These range shifts, however, are not consistently observed and vary greatly in strength and direction and are often species-specific^39^. For example, in the North Atlantic, some copepods including *Centropages chierchiae* and *Temora stylifera* were shown to move northward at a rate of 157–260 km per decade^37,40^. However, Chust et al.^14^ determined that movements of the copepod *Calanus finmarchicus* were an order of magnitude lower at 8–16 km per decade, whilst Edwards et al.^38^ showed that warming led to a decrease in krill abundance but no northward range shifts. In the Southern Ocean, poleward range shifts of Antarctic krill (*Euphausia superba*), the dominant zooplankton species, and salps have been recorded^41–43^, whereas the distribution of copepod species seems to have been resilient to warming of their habitat and remained fairly fixed^44^. Furthermore, range shifts of Antarctic krill were out of step with the pace of warming, with an abrupt shift occurring during a warming hiatus^45^. This nonlinearity was attributed to population processes, including an abrupt occupation of a new spawning ground in the south.

Species with low mobility (for example, planktonic foraminifera) can be more sensitive to environmental changes and water properties compared to mobile species (for example, euphausiids) that can modulate their spatial distribution to some extent. For example, global changes in temperature since the pre-industrial era have caused a range shift of ~40 km per decade of total foraminifera communities across the globe^46^. Furthermore, organisms inhabiting semi-closed basins (like the Mediterranean Sea) would not be able to shift poleward and may move to deeper layers, reflected as decreasing abundance trends in the surface warming waters^47^. These examples show that range shifts are often decoupled from the general poleward progression of isotherms^39,44^ and underscore the need for further studies to understand the mechanisms of climate change responses.

### Zooplankton size

Alongside shifts in phenology and range, declines in body size^21^ have been described as the third universal response to climate warming. Global studies on marine copepods, the most abundant multicellular aquatic animal on Earth, revealed that temperature was a better predictor of body size than either latitude or oxygen, with body size decreasing by 43.9% across the temperature range of −1.7–30 °C^22,23^. This suggests that with continued ocean warming, smaller copepod species are likely to dominate, with cascading effects on fisheries production and carbon sequestration^22,23^. This trend has not been observed in the Southern Ocean where average copepod community size shows a shift towards larger copepod species, but reasons for this difference are still unknown^48^. Within individual copepod species, adult body size was found to be generally reduced under warming (the temperature-size rule), but this temperature-dependence was recorded only during the spring to autumn growth period and was modulated by density-dependent effects from predation or competition^49^. Likewise, Antarctic krill showed a more complex size response under warming, where the changing balance of recruitment and mortality has led to a long-term increase in mean krill length^41^, opposite to predictions of reduced size with warming. Body size responses can be governed by factors beyond temperature or food, such as species behavioural and life history traits^50^, population dynamics^41^ or competition and predation^49^. Clearly, adjustments in body size for species or assemblages occur in parallel to shifts in range and phenology and they need to be studied within an integrated framework.

### Complex effects of climate change

Alongside the gradual and long-term change in global climate, marine heatwaves, defined as anomalously warm waters persistent for at least 5 consecutive days^51^, together with regional climate events (for example, El-Niño Southern Oscillation (ENSO), North Atlantic Oscillation (NAO), Southern Annular Mode (SAM); see Box 1), intensify observed trends in zooplankton phenology, range and size. For example, between 2014 and 2016, the northeast Pacific experienced two successive warming events — a marine heatwave and El Niño. The anomalously warm conditions in the upper ocean led to the California Current Ecosystem being dominated by gelatinous zooplankton, copepods and euphausiid species that were observed further north than their typical ranges^52–54^, which impacted predator-prey dynamics. The pelagic tunicate, *Pyrosoma atlanticum*, was a new arrival to the Northern California Current during the marine heatwaves of 2014–2016, and exerted considerable grazing pressure on local phytoplankton stocks^55^. The marine heatwave also resulted in changes to zooplankton size structure where a sharp decline in the body sizes of juveniles and adults of the euphausiid, *E. pacifica*, was observed in coastal waters of northern California^56^.

In the North Atlantic, NAO-dependent northerly winds increase on-shelf transport and recruitment of copepodites as they vertically migrate from deeper waters^57^. In the Northwest Atlantic, zooplankton responses to NAO are not uniform, with different copepod species exhibiting different responses to the NAO driven by their varying temperature preferences and traits, such as diapause ability (see Ref. ^58^ and references therein). For instance, *C. helgolandicus* (warmer water species, does not exhibit diapause) increases in abundance during positive phases and decreases during negative phases, whereas *C. finmarchicus* (cold-water species that does exhibit diapause) increases during negative NAO phases and decreases during positive phase^58^. In the Southern Ocean, negative phase SAM has been associated with increased phytoplankton biomass^59^, greater abundances of the ice krill *E. crystallorophias*^60^, and higher condition factor in mature female Antarctic krill^61^. In the Bering Sea, North Sea and northern Humboldt upwelling system, jellyfish biomass is positively correlated to SST, NAO and El Niño, respectively, as well as the availability of their prey^62–64^. Jellyfish ingest zooplankton, juvenile fish and fish eggs, and in the Bering Sea, jellyfish biomass peaked with moderate SST and low zooplankton biomass and moderate juvenile pollock biomass, but jellyfish biomass decreased with very warm temperatures, low zooplankton biomass and very high juvenile pollock biomass^62^.

Studies are increasingly highlighting the complexity and interrelatedness of responses to climate change. Aside from long-term ocean warming and regional climate events impacting phenology, range and size, to complicate matters further, climate change can have both direct effects on zooplankton through their biology, and indirect effects via their food. A combination of these effects was invoked to explain a pronounced and long-term, but summer-specific, decline in copepods across the Northeast Atlantic and fringing seas^65^. Warmer summers were suggested to have led to increased energetic demands for copepod metabolism, at the same time as leading to earlier spring blooms and a longer stratified and more nutrient-starved period favouring picocyanobacteria, which are too small to be ingested by most zooplankton^65^. Similarly, in the South-West Mediterranean, warmer and longer summers are suggested to drive the increased abundances of cladocerans in autumn, delaying the dominance of copepods towards the winter season^47^. Further studies are needed, particularly from the tropics and southern regions, to examine how multiple interacting factors influence the phenology, range and size of zooplankton, as this will undoubtedly have strong implications on ecosystem function.

### The biological carbon pump

**Fig. 2:**
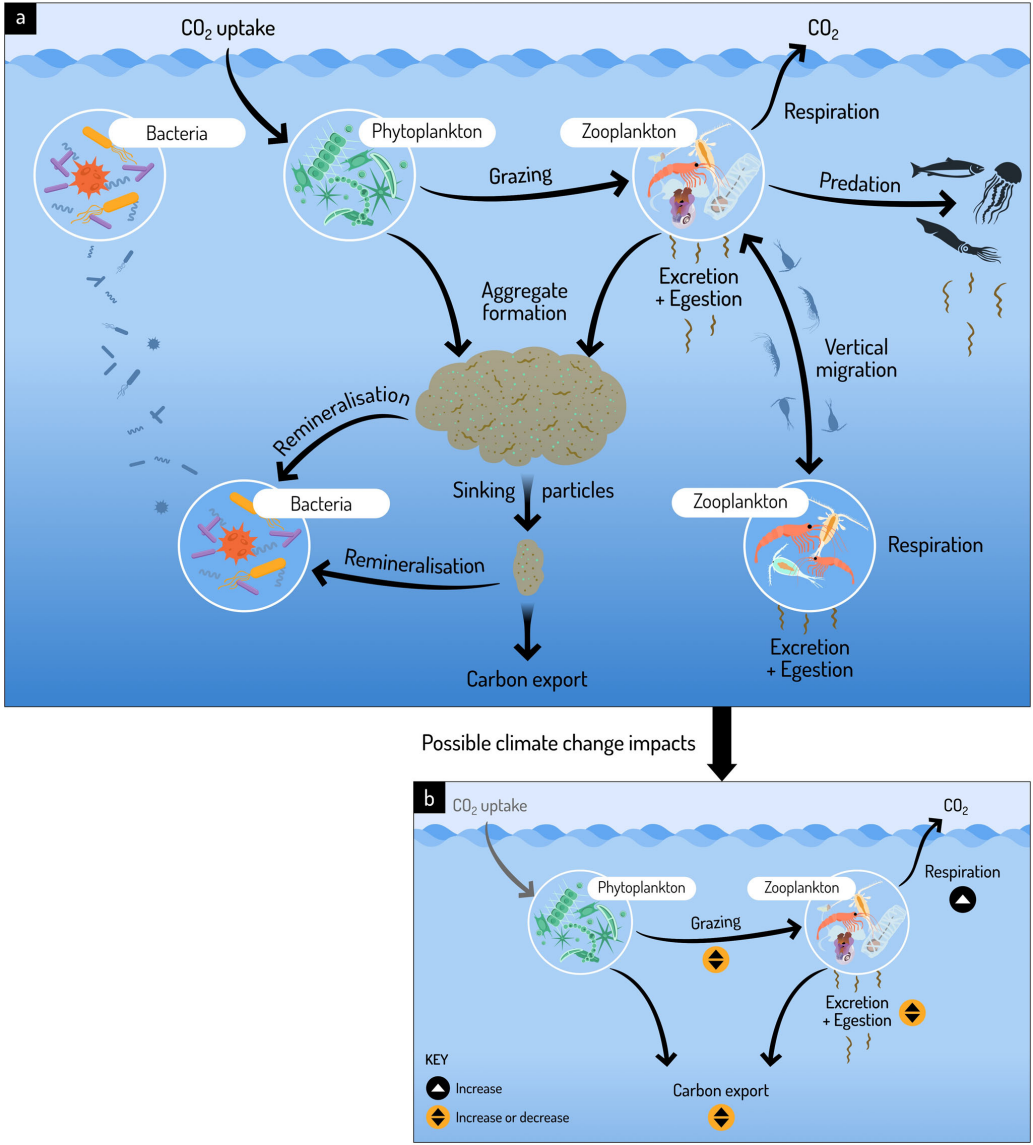
The role of zooplankton within the biological carbon pump and possible climate-driven impacts on key zooplankton processes. **a** Zooplankton graze on phytoplankton, transferring carbon and nutrients. Excess nutrients in zooplankton are recycled via excretion and egestion either within the upper ocean or throughout the entire water column as some zooplankton undertake diel vertical migration. Unconsumed phytoplankton form aggregates, and together with zooplankton faecal pellets, these particles rapidly sink and are exported to deeper waters. However, bacteria remineralise much of these sinking particles along its descent. **b** The smaller figure showcases the potential direction of change on three zooplankton processes – respiration, grazing, and excretion and egestion, under ocean warming. Studies to date show that zooplankton respiration will increase under a future warmer ocean, however the magnitude of grazing and excretion and egestion are unclear. Consequently, the magnitude of carbon exported through zooplankton-related activities under ocean warming remains unclear. This figure was designed by Dr Stacey McCormack (Visual Knowledge).

Zooplankton play a critical role in the biological carbon pump (Fig. 2). Through a series of transformations, zooplankton both recycle essential resources (for example, iron, dissolved organic carbon, ammonium, nitrogen and phosphorus) required for phytoplankton and bacterial growth, and export carbon to deeper waters^5,7–12,66–69^.

In the iron-limited Southern Ocean, iron is rapidly recycled by the copepod *C. simillimus*, Antarctic krill and the salp, *Salpa thompsoni*^7,8,12,66,70–72^, via grazing, excretion, egestion, vertical migration and bacterial remineralisation of sinking faecal pellets, which supports phytoplankton^12^ and bacterial production^6^. In contrast, at least one study has found that *S. thompsoni* faecal pellets deplete iron from Southern Ocean surface waters^73^, providing motivation for future studies on this topic^74^. Despite fewer studies in other oceanic regions, it is clear that zooplankton recycle a range of essential resources that support phytoplankton production (for example, iron and ammonium in the Atlantic Ocean^75,76^, nitrogen and phosphorus in the South Pacific^68^), but the few and conflicting studies highlight our uncertainties surrounding the magnitude and relative importance of various zooplankton groups^74^.

Zooplankton also contribute substantially to the cycling of carbon via respiration, sinking faecal pellets, sinking carcasses and diel vertical migration (DVM) across all oceanic basins (for example, Arctic Ocean^77,78^, Atlantic Ocean^79–83^, Pacific Ocean^84,85^, Southern Ocean^11,86–89^). However, there is large spatiotemporal variability with complex, and at times unclear, drivers. For example, the magnitude of the DVM-induced carbon flux depends on the species composition and biomass of zooplankton communities and can account for 4–70% of the total particulate organic carbon flux^90^. This active flux can exceed the passive sinking flux in the presence of mesoscale features (like eddies and fronts)^91^, whose weakening due to global warming would affect diel migrating and diapausing species biomass and downward fluxes^92^. Additionally, based on long-term Continuous Plankton Recorder (CPR) data, carbon fluxes over the last 55 years have increased along the northern and north-western boundaries from Iceland to the Gulf of Maine and decreased across much of the open northern North Atlantic and the European Shelf Seas^13^. Changes (and variability) in flux estimates could be due to changes in the distribution of copepod populations, related to the NAO which controls ocean currents and the advection of copepods, as well as the species-specific response in the distribution of plankton species to climate change^13^.

While ocean warming increases the energetic demands of zooplankton, it also increases stratification-driven nutrient limitation and leads to smaller zooplankton body size, which have negative effects on community production and carbon export^13,65,93^. Furthermore, large-scale climate oscillations drive shifts in zooplankton biomass and community structure^46,52–54,58,60,94–98^. So how will the role of zooplankton within the biological carbon pump change with ocean warming? Whilst there are many uncertainties, below we consider the direction of change for three key activities — respiration, grazing and excretion or egestion — that recycle and export nutrients and carbon, and drive a component of the biological carbon pump.

Respiration is a major loss term for organic carbon. Approximately 50% of the carbon ingested by zooplankton is respired, but this is strongly influenced by temperature and body mass^69^. The proportional increase in respiration per 10 °C rise in temperature (Q_10_) for zooplankton is on average ~1.9^69^ but this is variable. For example, salp (*S. fusiformis*) metabolic rates more than doubled with a temperature increase from 10 °C to 17 °C (1.66 to 3.95 μmol O_2_ g^−1^ h^−1^ wet weight, Q_10_ of 3.45^99^). In addition, while respiration increases with total body mass, weight-specific respiration decreases with body mass^69^. Thus, smaller sized zooplankton, as projected under a warmer climate, will have higher weight-specific respiration rates, leading to a greater loss of carbon (Fig. 2). Other factors that influence respiration rates include pressure, turbulence, oxygen, pH and feeding conditions (reviewed elsewhere^100^).

Grazing transfers nutrients and carbon from phytoplankton to zooplankton. Under a warming ocean, grazing pressure could increase or decrease (Fig. 2). Lewandowska et al.^101^ proposes a conceptual model of grazing pressure based on the prevailing nutrient regime. In oligotrophic conditions, phytoplankton growth is regulated by low nutrient supply, and zooplankton grazing is constrained^101,102^. In contrast, in eutrophic conditions, ocean warming influences plankton through metabolic changes, thus warming leads to an increase in grazing pressure and a decrease in phytoplankton standing stock^101^. Multiple mesocosm and modelling studies have pointed towards an increase in zooplankton grazing rates under a warming ocean causing a decrease in phytoplankton standing stocks because heterotrophic metabolism is more sensitive to temperature than autotrophic metabolism^102–105^. However, warming has also led to a decrease in zooplankton biomass which then reduces grazing and leads to greater carbon export^103,106^. Thus, direction of change for grazing rates could depend on the combined effects of temperature driven changes on metabolism and biomass, as well as the prevailing nutrient conditions.

Nutrients and carbon grazed in excess of demand are excreted and egested from zooplankton. We can examine if excretion and egestion will increase or decrease under a warmer ocean based on the stoichiometric imbalance of C:N:P ratio between predator (zooplankton) and prey (phytoplankton). If predator and prey stoichiometries are similar, then assimilation is optimal and recycling is low but departure from this optimal stoichiometry leads to a decrease in assimilation efficiency and an increase in nutrient recycling^107^. Healthy natural assemblages of marine plankton often tend to have molar C:N:P ratios of around 106:16:1 (Redfield ratio), but this ratio can vary depending on environmental conditions. The ratio is higher in oligotrophic subtropical gyres (~195:28:1) and lower in eutrophic polar waters (~78:13:1)^108^. There is increasing evidence that phytoplankton elemental stoichiometry varies with environmental conditions, physiological demand and evolutionary factors (for example, luxury iron uptake to counter iron deplete oceanic waters)^109^. It is unclear how zooplankton stoichiometry will change under warming and, consequently, the magnitude and direction of nutrient cycling based on predator-prey stoichiometry is uncertain.

In summary, aside from zooplankton respiration, which is expected to accelerate under ocean warming, the direction of change for grazing and excretion and egestion are unclear. As we layer these uncertainties with those of zooplankton phenology, range shifts and size, particularly noting the paucity of studies with at times contradictory findings from the Southern Hemisphere, we are unable to predict how zooplankton will modulate the biological carbon pump under future conditions. Complex multi-driver experiments in a fully factorial matrix can quickly become logistically impractical. To combat this challenge, designing multi-driver experiments with variables that reflect local or regional settings (for example, current and projected changes in SST, or phytoplankton community structure, quality and quantity, amongst others) can be an important and informative step to discern emerging patterns and guide model parameterisation and validation.

### Interactions with higher trophic levels

Zooplankton act as a conduit for the transfer of energy from phytoplankton to higher trophic levels, including commercially important fisheries — an industry estimated to be valued at US$401 billion in 2018^110^. Evidence is mounting that the phenology of lower trophic levels (phytoplankton, zooplankton) is moving 5–10 days earlier per decade, faster and more consistently than higher trophic levels (adult fish, seabirds, marine reptiles and mammals) that are moving earlier by 0–2.5 days per decade^29^. This contrasting response could lead to trophic mismatch, whereby the timing of predators and their prey responds asynchronously to climate change^18^, with potential ecosystem consequences including poorer fish recruitment^111,112^, altered fish migration^113–115^ and changes to the spawning of fish^116,117^, crabs and squid^118^.

Whilst overfishing can set pressures on fish stocks, below we showcase how the survival of larval fish also depends on the mean size, seasonal timing and abundance of prey^119^. In the Sea of Japan/East Sea region, increased squid catches were attributed to increases in zooplankton biomass, particularly euphausiids and amphipods^120^. In the North Sea, reductions in euphausiid and copepod size and abundance has led to the decrease in cod recruitment since the 1980s^119^. In the Straits of Georgia and more broadly within the Northern California Current, lower zooplankton biomass resulted in the impoverished growth and survival of juvenile salmon and herring^121,122^. In the Western Mediterranean Sea, it is suspected that changes in the zooplankton communities might be behind the decline in European sardine and European anchovy stocks^123^. Given that these species and their larvae prey on the most abundant copepods in the region^124^, changes in the composition and distribution of their prey (notably lipid content^125^) could affect the condition and success of these small pelagic fishes with important socioeconomic consequences, and therefore concomitant monitoring of plankton and fishes is recommended to elucidate the relationship between zooplankton variability and fisheries success^126^. There have been increasing efforts to develop ecosystem based management of fisheries; however, fisheries evaluations or models typically include oceanographic and chlorophyll (derived from satellite sensors) data as variables and not zooplankton biomass or abundance, despite the importance of zooplankton in understanding the transfer of energy to fish and fisheries^127^. Further cooperation between fisheries and plankton experts is needed to understand the type of zooplankton data and traits needed by modellers to include this crucial component of the trophic web in fisheries management models.

Changes in zooplankton distribution can also influence top predators; however, elucidating direct relation to zooplankton is difficult due to the complex structure of oceanic food webs involving multiple trophic levels. Around the California coast, fewer, high nutritional quality euphausiids due to the 2014–2016 marine heatwave resulted in a shift in the foraging behaviour of whales, which contributed to increased rates of their entanglement^128^, whilst seabird populations also experienced unprecedented die-offs in the same location^129^. Similarly, off the Northeast coast of the US and the Gulf of Maine, there have been declines in Northern Right Whale preferred prey species (*C. finmarchicus*), causing range shifts in the whales, leading to increased entanglements and ship strikes, and negatively impacting calf mortality rates^130^.

In the Southern Ocean, seal diving patterns reflect the cascading vertical distribution of prey^131^. Specifically, sea ice break-out stimulates a strong resource pulse of phytoplankton in the Ross Sea which then triggers cascading changes in the vertical distribution of zooplankton, fishes and seals^131^. Although interpretations of the response of higher trophic levels to climate driven changes in zooplankton are difficult, coupling long-term physical, biogeochemical and biological observations can shed light on the synchrony of timing between primary, secondary and tertiary production, and ultimately ecosystem function. Incorporating these observations into models could provide strategic insight that enables predictions of future climate-driven changes across spatial and temporal scales, which is not possible from observations alone. However, models require a range of information to improve parameterisation and validation, as described below.

## The challenges of modelling zooplankton in a changing climate

Models provide a powerful method that extends inherent limitations of field and laboratory experiments to improve our understanding of marine ecosystems under a changing climate^132–134^. Many models describe plankton organisms based on their multiple functionalities (for example, coccolithophores are represented as autotrophs, calcifiers and prey) and come in various designs and levels of complexity, such as a simple food chain of Nutrient–Phytoplankton–Zooplankton to complex microbial food web with bacteria, autotrophs, mixotrophs and heterotrophs^132^. Despite advances, zooplankton are misrepresented in most numerical models^132,135^ due to the complex life cycles of mixotrophs and heterotrophs (especially metazoans), the complex species-specific behaviour of mesozooplankton and the costly field and laboratory observations that limit our knowledge to a few dominant species mostly from ciliates, copepods and krill.

Ultimately, the gap between what is observed versus modelled coupled with over simplistic representation in models due to the lack of mechanistic constraints, reduces our confidence in model projections. Even small changes in zooplankton parameterisation can have significant effects on projected population dynamics, community structure and energy transfer^4,136,137^. Empirical data needed for model parameterisation, comparison and validation can be categorised in three main components: (1) rates, such as ingestion, respiration, growth, reproduction, excretion and egestion; (2) traits, such as size, foraging, diet breadth, reproduction and stoichiometry; and (3) stocks, such as spatial and temporal coverage of biomass and abundance of various types of plankton. The interactions between these three components directly control how zooplankton influence biogeochemical processes, thereby driving the need of integrating these parameters within biogeochemical models.

For models that parameterise species ecophysiology, qualitative information exists for most zooplankton traits such as body size, foraging, diet, reproduction and predation^138^. However, the lack of quantitative information on individual species and community trade-offs limits our understanding of their dynamic response to environmental stimuli. For example, many copepods can rapidly switch their foraging strategy in response to prey availability and predator presence^139^, and recent studies suggest opportunistic foraging by zooplankton that indicate their diet breadth is greater than previously considered^140–143^. New observational insights obtained regarding zooplankton predator–prey interactions and DNA metabarcoding (individual organisms and water) revealing diet composition can provide greater detail on diet breadth^144^, which will improve grazing parameterisations in models.

When it comes to stocks, most models describe individuals or populations in terms of biomass. Unfortunately, most of the global zooplankton biomass estimations are of sample biomass (that is, the biomass of all zooplankton and non-zooplankton included in the sample). The lack of empirical data expressed in useful formats for models leads to unequal comparisons between model and observations and delays on model improvements. For example, complex life historical models for metazoans^145,146^ or ecosystem models with various zooplankton size groups^147^ lack empirical data in forms that will enable a direct model–observation comparison for validation and further advancement. Key to overcoming this barrier is the use of image analysis methods that deliver trustworthy biomass calculations based on individual body properties, such as size-dependent and taxa-dependant allometric biomass to volume conversion factors^148^. Recent development of in situ imaging technologies^149,150^ and recognition algorithms^151^ deliver zooplankton biomass distribution for different taxa and sizes in a currency consistent with models much faster compared to sampling and taxonomic determination under microscope^151^. Additionally, a thorough examination of the many existing conversion equations for estimating biomass from abundances or biovolume^152^ are needed alongside the development of a universal conversion table agreed by the international community.

Additionally, zooplankton biomass and rates in most biogeochemical models are restricted to the epipelagic layer, due to the lack of knowledge and data from the mesopelagic and bathypelagic regions (both spatial and temporal). Consequently, active fluxes driven by zooplankton vertical migration are disregarded in the models. Insights on mesopelagic and bathypelagic zooplankton communities and DVM will improve quantitative knowledge on zooplankton contributions to the biological pump and nutrient recycling. Together, these advances will enable us to quantitatively explore key scientific questions at regional and global scales. For example, what are the processes that drive species movement or displacement as observed in the North Pacific (poleward migration^37,40^) and Southern Ocean (poleward migration^41^ and possible displacement of Antarctic krill by salps^42^)? What are the implications of such movement or displacement on biogeochemical processes and/or trophic interactions? In a broader sense, to project the impacts of ocean change on regional fisheries production, higher trophic levels and the biological carbon pump with confidence, zooplankton needs to be fully integrated in various modelling frameworks. Whilst enormous progress has been made through various monitoring programmes and networks, our fragmentary picture based on observations largely from the Northern Hemisphere, is not sufficient.

## Sustained observations to quantify impacts of climate change

Untangling natural from anthropogenic change across temporal scales (for example, long-term due to global climate change versus multi-annual fluctuations due to regional climate forcings such as El Niño or the NAO) requires sustained observations over many decades^106,153,154^. Yet most funded projects run for 3–4 years and funding for long-term research is in decline^155^. LTER, defined as studies in which ecological data have been collected regularly and systematically from a site, or set of sites, over a period of more than 10 years^156,157^, enables us to: quantify ecological responses to environmental change, including climate change; understand complex multi-year ecosystem processes; develop theoretical ecological models and parameterise and validate simulation and management models; serve as collaborative platforms promoting multidisciplinary research; and support evidence-based policy, decision-making, and management^158^.

For example, in the Eastern Bering Sea, long-term oceanographic research has identified ecosystem regime shifts that oscillate in response to multi-year variability in the size of the Eastern Bering Sea cold pool^94–96,98^. The Palmer LTER project in Antarctica has demonstrated connectivity between ecosystem productivity at the Western Antarctic Peninsula and the climatological indices of the SAM and ENSO^59–61^. Similarly, in Brazil, the Brazilian LTER at the estuary of the Patos Lagoon and adjacent coast revealed that composition of phytoplankton, zooplankton, benthic flora and macrofauna were affected by different scales of variability related to ENSO^97^. In the Mediterranean Sea, a meta-analysis of several time series over the last 50 years highlighted substantial changes in plankton community composition that resulted from direct local anthropogenic nutrient input or basin-scale decadal evolution related to the NAO^159^. When Northern Hemisphere time series data were aggregated, a regime shift (large, persistent change in the state of the community or ecosystem) was identified in the 1980s due to an increase in Northern Hemisphere air and SST and a strongly positive phase of the Arctic Oscillation; however, there was considerable regional variability^160^.

**Fig. 3:**
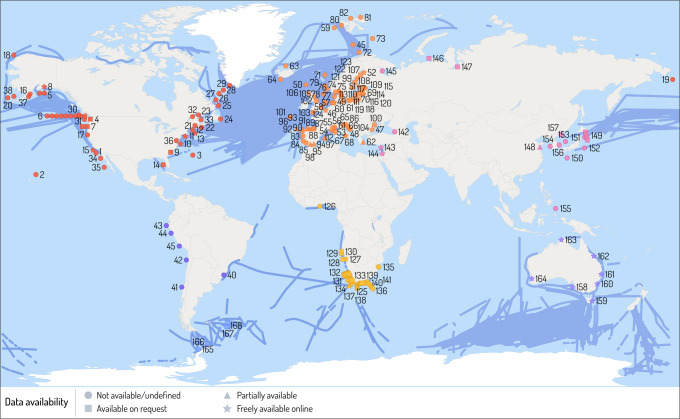
Map of long-term monitoring programmes for zooplankton in the global ocean. Blue lines indicate Continuous Plankton Recorder (CPR) surveys and symbols indicate sites of specific long-term monitoring programmes (see Supplementary Data 1 for details of numbered sites). Stars indicate data is freely available to download, squares indicate data available on request, triangle indicates partially available, and circles indicate data either not available or unclear on data availability. Only programmes where coordinates were available were plotted. Data sourced from the Marine Ecological Time Series Database, EuroSea survey and surveys undertaken as part of this review effort. More information and coordinates are provided in the Supplementary information. This figure was designed by Dr Stacey McCormack (Visual Knowledge).

Complex physical, biogeochemical and biological processes interact to shape a given region; thus, scaling local and regional findings into a global context is likely to be non-linear. Additionally, relatively few multi-decadal long-term studies and datasets exist for ocean ecosystems^161^. We identified 168 long-term zooplankton monitoring programmes and CPR surveys undertaken in 6 oceanic regions (through the Marine Ecological Time Series Database^162^, EU Horizon 2020 EuroSea survey^163^ and surveys undertaken as part of this Review). In Fig. 3 (see also Supplementary Data 1), we separate the 168 long-term zooplankton monitoring programmes and 6 CPR survey regions because, while they both sample zooplankton, CPR surveys have a much wider spatial coverage and the CPR also surveys for large phytoplankton. Of all these monitoring programmes, ~19% had their data freely available, 9% had data that partially available (that is, part of the data was available and part was restricted or unavailable for various reasons), data for 13% were available on request, data for 7% were not available and ~52% were undefined (unable to determine data availability) (Fig. 3). Of the programmes that had their data publicly available, zooplankton were sampled using different techniques and the data were stored in various repositories, thus identifying comparable descriptors is challenging.

An overwhelming 81% of the data collected from long-term monitoring programmes is either partially available or not publicly available, which prevents the scientific community from answering large-scale questions about the response of zooplankton to climate variability and long-term climate change. Renewed effort is needed from the research community, funders and journals alike to ensure that crucial long-term monitoring data, particularly on zooplankton abundance, biomass and diversity required to understand phenology and range shifts, is made publicly available for global analysis to be undertaken. We provide two examples that highlight the success of open-source data. The first is the recent introduction of jellyfish into the PlankTOM11 model^164^ using observational data extracted from the MAREDAT database^165^. Modelling jellyfish abundance against observed data provided confidence in the model results, including the important role jellyfish have in regulating marine ecosystems, particularly in controlling macrozooplankton biomass and its cascading impact through the ecosystem^164^. A second example is the inclusion of zooplankton data from the COPEPOD database^166^ to produce robust global maps of zooplankton biomass and abundance of different functional groups used to test a global zooplankton size-spectrum model and the possible effects of changes in zooplankton on fish stock^146^.

Aside from national monitoring programmes, regional and global networks that foster international collaboration and undertake sustained observations across national boundaries do exist within the zooplankton scientific community. However, there are still large regions, especially in the open ocean or areas beyond national jurisdiction, which remain uncovered by this collaborative structure (Fig. 3). Regional and global zooplankton networks include the Global Alliance of Continuous Plankton Recorder Surveys (GACS), the International Council for the Exploration of the Sea (ICES) Working Group on Zooplankton Ecology (WGZE), the North Pacific Marine Science Organization (PICES) working groups focussed in the Pacific region, the Working Group on Mediterranean Zooplankton Ecology (MedZoo.bio), and the Integrated Marine Observing System (IMOS) National Reference Stations focussed within the Australian region. Although CPR surveys (blue lines, Fig. 3) play a key part in surveying much of the North Atlantic, the northern reaches of the North Pacific and parts of the Southern Ocean, large gaps exist across the rest of the global oceans, particularly the South and Equatorial Pacific, Indian Ocean, South Atlantic and the Arctic Ocean. Long-term monitoring programmes (points, Fig. 3) are well represented in coastal Australia, Europe, South Africa, and North America, but large gaps exist in coastal Asia, South America and much of Africa. Long-term monitoring programmes are also severely lacking in offshore, open ocean locations. Moving forward, we must fill these gaps, either through establishing long-term monitoring programmes and/or developing new technologies that facilitate data collection in these remote locations.

## The future of global zooplankton research

As highlighted above, ocean warming is impacting zooplankton phenology, range, and size, with  flow on impacts on the biogeochemical cycles and transfer of energy and matter to higher trophic levels. There are key observation and modelling knowledge gaps as well as observational data coverage that need urgent address. In the following subsections we discuss how different technologies can be used together to improve knowledge and geographic coverage, and how the data obtained can be used to meet global needs.

### Filling the gap with an integrated approach to zooplankton research and monitoring

There have been several recent reviews on modern plankton sampling techniques and observing systems^149,167–169^. Here, we highlight how an integrated approach using established and new technologies can elucidate the response of zooplankton to climate change. Nets (for example, bongo, WP2, ring, neuston, rectangular midwater trawl) with various mesh sizes capture different zooplankton groups and enable analysis of species abundance, composition, community size structure and spatial distribution. However, limitations of using nets include their depth integrative nature, potentially destructive collection mode toward delicate organisms, and poor sampling of some taxa such as gelatinous zooplankton^132^. The increasing need for zooplankton data, limited budgets for monitoring, and the potential for gaining new insights into different aspects of zooplankton dynamics has driven the development of new technologies for collecting zooplankton information. Newer technologies such as the Continuous Automatic Litter and Plankton Sampler (CALPS^167^), automatic sensors using acoustic (Acoustic Doppler Current Profilers (ADCP) or echosounders), and in-situ imaging (for example, PlanktoScope^170^, Imaging FlowCytobot^148^, Plankton Imager^171^, Zooglider^172^ or Underwater Vision Profiler (UVP)^173,174^) can sample large ocean areas, have high vertical and horizontal resolution sampling capacities, but low taxonomic resolution compared to manual analysis of samples via microscope. Although novel molecular methods (such as eDNA, eRNA, (meta)genomics, (meta)barcoding, (meta)transcriptomics) allow for unprecedented taxonomic identification capabilities and qualitative description, they suffer from a lack of established reference samples, and as yet unproven quantitative rigour, but they are steadily improving. There are trade-offs associated with the choice of sampling method, and selecting a method depends on the scientific question and available budget. It is important to note that time series length is a key determinant of its statistical power to detect change^175^. Thus, the many existing time series based on traditional methods should not be replaced by new methods unless they provide nearly identical information. Instead, combining traditional tools with new technologies and omics can open new horizons on the type of data collected, and this can provide a mechanistic understanding of species behaviour (for example, adaptation or trade-offs) and rates. The data can also be used for model parameterisation and validation to advance our forecasting tools and policy decisions.

**Fig. 4:**
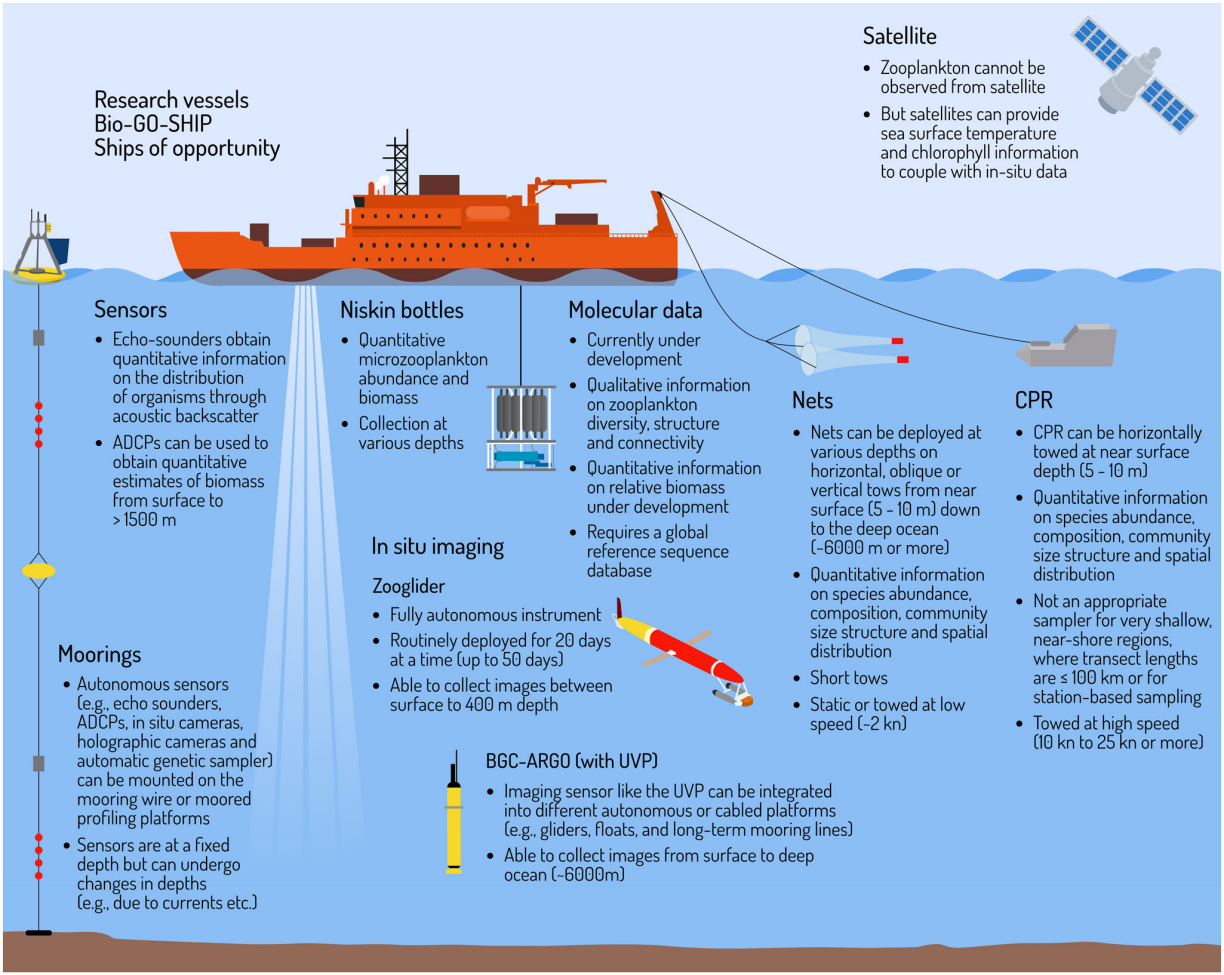
Integrating traditional zooplankton in situ sampling methods and modern sampling techniques. Traditional techniques such as Continuous Plankton Recorder (CPR), nets and Niskin bottles have been used to monitor zooplankton for decades with great success. However, coupling traditional techniques with newer methods such as molecular data (for example, DNA, RNA and proteins), advanced sensors, in situ imaging approaches and satellites can improve geographic coverage, particularly in under sampled regions and improve our understanding of the impact of climate change on zooplankton communities. Whilst the CPR, nets and Niskin bottles are shown together, they are not generally conducted simultaneously. This figure was designed by Dr Stacey McCormack (Visual Knowledge).

The increasing variety of sampling devices and strategies used prevents homogeneous and inter-comparable data and provides a challenge for co-designing an integrated approach. However, to address the cross-disciplinary questions posed in this review, we need an integrated approach allowing cross comparison and merging between the various traditional and modern techniques. For example, qualitative eDNA is largely used for biodiversity estimates, but ground-truthing eDNA data with quantitative data from nets can improve our monitoring of spatial and temporal variation in zooplankton structure. Similarly, imaging platforms (such as Zooglider) provide quantitative data on zooplankton distribution at higher spatial resolution than nets^172^. Coupling nets with imaging and eDNA enables us to gain innovative insights into zooplankton community structure at finer scales over larger distances. To progress this further, combining zooplankton data with physical and biogeochemical characteristics (for example, from biogeochemical ARGO floats, CTDs or satellites) can provide observational insight on how zooplankton are impacted by the environment. This information constitutes the basis of mathematical and numerical analysis. In addition to mathematical modelling, recent machine learning methods can be used to model zooplankton distribution from environmental data^176^. Incorporating mathematical and numerical modellers at the onset of such studies can both improve model parameterisations and expand spatial and temporal extrapolations of observational data. Such efforts have been performed in several studies to investigate the Arctic zooplankton communities with in situ observed and net–collected plankton^177^, in mesopelagic layers by combining imaging systems, nets and BGC ARGO floats^178^, in the Celtic sea to compare an imaging system with automated sample collection and traditional vertical ring net vertical deployment^171^, and in the USA through the Bio-Global Ocean Ship-based Hydrographic Investigations Program (GO-SHIP)^179^. These studies merging various traditional and modern techniques are prototypes for a global integrated approach and demonstrate that the oceanographic research community can work together to increase the broader application of their individual information (Fig. 4).

Two key pathways forward are to increase monitoring efforts in prioritised under sampled regions and undertake collaborative sampling and experiments. For the former, the deployment of a network of ARGO floats with UVPs, or other novel technologies, in remote locations as well as poorly monitored coastal areas can help fill this geographic and knowledge gap but this will need to be supported with more detailed local sampling to build an understanding of local taxonomy and phenology (Fig. 4). Ships of opportunity could also be more widely used to expand CPR routes and include automated tools (Fig. 4). For the latter, stronger collaborations are required between zooplankton ecologists, and other biological and biogeochemical oceanographers, as well as modellers, to identify the key parameters and processes that need quantitative information. These collaborations, if formed from the onset, will lead to novel multi-driver experimental-modelling approaches, and ensure that all components of the system being examined are thoroughly thought through from data collection to the delivery of interpreted information.

### Linking zooplankton observations to global needs

Tracking how marine life responds to increased human use and climate change will empower the global community to understand, predict, protect and interact sustainably with our ocean. The global observing community recognises the need to expand the continuous, long-term observation of marine life, and to fill gaps where these observations are lacking. The biology and ecosystem Essential Ocean Variables (EOVs) — of which zooplankton biomass and diversity is one — represents one approach to coordinating the global ocean observing community^180^. The EOVs and requirements for global marine life observations are being advanced by the Global Ocean Observing System (GOOS) in partnership with the Marine Biodiversity Observation Network (MBON) Essential Biodiversity Variables (EBVs) and the Ocean Biodiversity Information System (OBIS). The aim is to ensure open data sharing that aligns with and facilitates global marine biodiversity assessments. The biology and ecosystem EOVs are also a means for reporting on biological Essential Climate Variables to the Global Climate Observing System (GCOS), where zooplankton is also included.

Information on trends in diversity, distribution, and abundance of zooplankton will also contribute to Sustainable Development Goal (SDG) 14 (life below water), the Convention on Biological Diversity post-2020 Agenda, and World Ocean Assessments, among other mechanisms. Progress on coordinated zooplankton observations as a contribution to this global imperative will require expert communities coming together to address gaps in observing by supporting increased long-term monitoring, including in under sampled regions. To that end, we propose four key steps forward to meet global needs: (1) Protect existing, and build new, time series programmes; (2) Better integrate time series data; (3) Broaden our understanding of climate change responses; and (4) Improve cross-disciplinary approaches (Box 2). The results of improved integration of sampling, modelling, and reporting activities will lead to increasingly rapid understanding of the dynamics of zooplankton communities at regional and global scales, their likely response to ongoing climate change, and the implications that this response and its regional variability will have on local resource production and global ecosystem services. Such improved understanding will benefit the research community and could address societal needs.

Box 2 Towards an improved implementation of zooplankton monitoring to address global needs

**Table:** 

Protect existing and build new time series programmes	Time series can be difficult to establish due to lack of long-term funding, lack of widespread understanding of the importance of long time series to the study of climate change-scale processes, and pressures for monitoring programmes to adopt new technology. Even subtle changes (for example, a slight change in net design) require a lengthy parallel intercalibration period to ensure comparability. Additionally, as highlighted in Fig. 3, there are large gaps in coastal Asia, South America and much of Africa, and offshore, open-ocean regions where monitoring is crucially needed.
Better integrate time series data	Efforts are needed to actively engage with monitoring programmes as well as regional and global networks to better integrate time series data. This includes making existing data more easily available, encouraging group efforts to synthesise data across multiple time series, and ‘rescuing’ and combining old data which will allow for large spatio-temporal studies to understand climate change responses.
Broaden our understanding of climate change responses	Existing understanding is relatively unbalanced, often dominated by productive, mid latitude shelf ecosystems, and mostly of adult stages of dominant taxa. Copepoda is the most studied zooplankton group with the literature being focused on a few dominant species (for example, many of the *Calanus* species and the coastal species *Acartia tonsa*). Modern technology (for example, moorings, acoustics, molecular approaches, particle imaging) can be used to increase observations in poorly sampled systems, and address issues such as extreme events (responses to heatwaves or storms). A key focus should also extend observing and data collection efforts for holo- and meroplanktonic taxa (including small or gelatinous forms).
Improve cross-disciplinary approaches	Time series data, experimentation, and modelling in combination can provide a powerful approach to understand the mechanisms that zooplankton use to adjust to recent climate change. Particularly experiments that examine multiple climatic stressors, as this will enable better understanding of how the biological carbon pump and food web structure may be impacted. Likewise, engagement of zooplankton ecologists with working groups on resource management (for example, fisheries or conservation zones) and policy can ensure the data products are well utilised and will support the continuation of monitoring.

## Supplementary information

Description of Additional Supplementary Files

Supplementary Data 1
