# Supplementary material for: Monitoring and modelling marine zooplankton in a changing climate
Source: Nat Commun. 2023 Feb 2;14:564. doi: 10.1038/s41467-023-36241-5 (PMC9895051; doi:10.1038/s41467-023-36241-5)
Supplement: Supplementary file 1 — Description of Additional Supplementary Files [file 41467_2023_36241_MOESM1_ESM.pdf]

Description of Additional Supplementary Files

File Name: Supplementary Data 1

Description: Information on long-term zooplankton monitoring programs in the global ocean
